# A 34-year overview of night work by occupation and industry in France based on census data and a sex-specific job-exposure matrix

**DOI:** 10.1186/s12889-022-13830-5

**Published:** 2022-07-29

**Authors:** Marie-Tülin Houot, Nastassia Tvardik, Emilie Cordina-Duverger, Pascal Guénel, Corinne Pilorget

**Affiliations:** 1 Santé publique France, The French Public Health Agency, 12 rue du val d’osne, 94415 Saint-Maurice, France; 2 Center for Research in Epidemiology and Population Health (CESP), Team Exposome and Heredity, Inserm, Université Paris-Saclay, Institut Gustave-Roussy, 94807 Villejuif, France

**Keywords:** Night shift work, Job-exposure matrix (JEM), Exposure prevalence, Occupational exposure, Trend, Exposure proportion

## Abstract

**Background:**

Night work has been increasing in the last decades due to new working arrangements for good and services production. Numerous studies have shown that night shift work causes disruptions in circadian rhythms that may affect health. In 2019, night shift work was classified as probably carcinogenic to humans by the International Agency for Research on Cancer, and may contribute to other health disorders. In this context, we assessed the number and proportion of workers exposed to night work today and investigated time trends by occupation and industry in France since 1982 in terms of prevention.

**Methods:**

Using the data on work time schedules collected in the French Labour Force Surveys, sex- and period-specific job-exposure matrices (JEMs) to night work (working between midnight and 5 AM) were developed. After linkage of the JEMs with data of the national censuses of 1982, 1990, 1999, 2007 and 2015, the numbers and proportions of workers usually or occasionally exposed to night work were estimated.

**Results:**

The number of night workers (usual and occasional) increased from 3.67 million in 1982 to 4.37 million in 2015 (15.8% vs 16.4%). Night work was more common in men than in women (e.g. 22.4% vs 10.0% in 2015), and usual night work largely increased after 2000 (4.4% in 1999, 7.2% in 2007). In 2015, 1.29 million men worked usually at night, including 882,000 workers in the service sector (63%) and 360,000 in the manufacturing and extracting industries (28%). For the same period, 581,000 women were usual night workers, most of them being employed in the service sector (90%). Among women, a 97% increase of usual night work was observed between 1982 and 2015.

**Conclusions:**

This study shows that night work involves a growing number of workers in France, particularly in women in the service sector. These results raise concern about the public health impact of night work and particularly about the numbers of outcomes attributable to this exposure such as breast or prostate cancers.

**Supplementary Information:**

The online version contains supplementary material available at 10.1186/s12889-022-13830-5.

## Introduction

Several occupations have traditionally been carried out both day and night, such as those that require 24 hours services for health care or security. The need for working-time arrangements that allow goods and services to be produced 24 hours a day, 7 days a week has increased over the last decades. Shift work typically involves working outside the standard daytime hours — such as at night, during evenings, or starting on early morning — and shifts can be permanent, switching from day to night, or without any particular pattern. Night shift work is a common occupational exposure, with approximately 19 to 25% of all workers in Europe and the United States working a variety of night shift schedules [1, 2].

Numerous studies have shown that shift work, in particular night shift, causes disruptions in circadian rhythms that may affect well-being and health [3]. Exposure to light at night (LAN) can lead to misalignement of the central biological clock with the day-night cycle, that contributes to sleep changes and circadian disruption [4]. Moreover, shift work affects multiple daily activities such as eating, sleeping, physical activity, tobacco smoking and alcohol consumption. These exposures can vary depending on the studied health outcome (sleeping disorders may be more important for fatal driving accident and co-exposures for cancer outcomes) [3]. Based on a literature review in 2019, the IARC monograph concluded that night shift work was probably carcinogenic to Humans (group 2A) [3].

In France, up to 2001, due to its legislation, women were not allowed to work at night except for some very specific activities such as industries processing perishable goods and in hygiene or well-being activities. Since then and to be in compliance with the European law based on the principle of gender equality in the workplace, the French labour code has been modified to allow night work to women and pointed out the derogatory nature of night work implying an enhanced medical surveillance of night workers [5, 6].

Exposure assessment in epidemiological studies may require the use of job-exposure matrices (JEMs) when studies involve large populations [7, 8]. For night shift work, the assessment is often derived from national labour surveys which provide sociodemographic data for the working population or from studies by interviews on a random of population [9–12], and similarly for existing JEMs [13, 14].

In order to provide information on exposure to night work useful for occupational exposure surveillance, our objective in this paper was to present estimates of the number and proportion of workers exposed to night work in France, overall and by occupation and industry, and study their evolution over 34 years using census data and sex-specific job-exposure matrices.

## Methods

The numbers and proportion of workers exposed to night work were estimated between 1982 and 2015 by sex and period by linking job-exposure matrices (JEMs) with occupational census data for the French population.

### Development of the JEMs

A series of JEMs, tables reporting proportion of exposed workers for each job (occupation in an industry), were elaborated from the data on work time schedules collected in the French national Labour Force Surveys (*“Enquête Emplois”*). These surveys have been conducted annually since 1993 and quarterly continuously over the year since 2003 by the National Institute of Statistics and Economic Studies (INSEE) to provide information on employment status in France [15–18]. The surveys conducted among individuals over 15 years of age living in randomly selected households, included 110,000 to 150,000 individuals per year.

For each subject, the Labour Force surveys provide information on age, sex and current occupation and industry coded according to the Professions and socio-professional classification (“*Profession et Catégorie Socio-professionnelle*”, PCS) and French industries classification codes (“*Nomenclature des activités française*”, NAF), respectively [19–23]. Each subject was classified as a usual night worker, an occasional night worker or a non-night worker, based on the answer to the question: “*Do you work at night, i.e. between midnight and 5:00 AM?*: *(1) yes usually, (2) yes only some nights, (3) never”* in a specific block on the main job in the surveys from 1993 to 2002, or “*In your main job, how often do you work at night, i.e. between midnight and 5:00 AM?: (1) usually; (2) occasionally; (3) never”* in the surveys from 2003 to 2012 (Additional file 1). Because of significant changes in the wording of the questions on work time schedules after 2012, the categorization into usual or occasional night workers could not be applied using the subsequent surveys which were not used in the present analysis (see Additional file 1).

We developed a series of JEMs for men and women separately combining the survey data by 5-year periods (1993–1997, 1998–2002, 2003–2007, 2008–2012). This chronological breakdown coincides with the different versions of the national PCS and NAF classification system, and takes into account changes in the wording of the questions on night work (Additional file 1). The JEMs (PCS x NAF JEMs) were elaborated in a flexible way by combining the PCS codes at a 4-digit level (PCS-4) and the NAF codes at a 2- or 3-digit level (NAF-2, NAF-3). The probabilities of being a usual or an occasional night worker in the PCS x NAF JEMs were calculated as the proportion of usual and occasional night workers in each job defined by the combination of PCS-4 and NAF-3 codes under the following two conditions: (i) the job included at least 30 individuals, and (ii) the precision of the proportion of usual night workers did not exceed 10%. If these conditions were not met, jobs were aggregated by combining PCS-4 with NAF-2 instead of NAF-3 codes. For the occupations poorly represented in the Labour Force Surveys that could not meet the conditions above, the probabilities of exposure to usual or occasional night work were calculated by PCS codes only regardless of the NAF codes using a “PCS-only” JEM. A complete description of the JEMs development is provided in Additional file 1.

### Statistical analysis

The sex- and period-specific JEMs were linked with the Census occupational data for metropolitan France (defined as European territory of France) using the sex, PCS and NAF codes as the matching variables. The census data from 1982, 1990, 1999, 2007 and 2015 were used [24, 25]. The 1982 and 1990 censuses were merged with the 1993–1997 JEM, the 1999 census with the 1998–2002 JEM, and the 2007 and 2015 censuses with the 2008–2012 JEM.

**Fig. 1:**
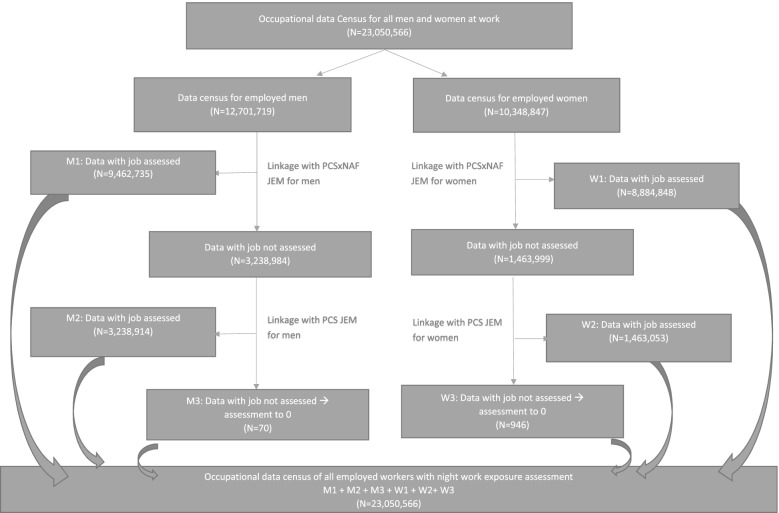
Example of linkage between the 1999 Census and the night work JEMs for 1998–2002 period

For each of these censuses, as presented in Fig. 1, the exposure assessment to night work was undertaken in two consecutive steps. First, the assessment was based on the detailed JEMs (PCSxNAF JEM), and taking into account the exposure period and sex. Secondly, for the job that would not have been assessed by the PCSxNAF JEM (certain jobs are not assessed due to lack of power), the exposure probability was assigned using the sex-specific PCS JEM assessing night work based only on the PCS. As the JEMs were sex-specific, the different exposure probabilities for men and women were considered at the time of the linkage (Fig. 1).

The numbers of usual and occasional night workers in France were obtained by multiplying the exposure probability provided by the JEMs by the number of workers in the job in the census data. The proportions of usual and occasional night workers were obtained by dividing the total number of usual and occasional night workers by the total number of workers in the population. A sensitivity interval (SI) for the proportion of exposed workers was calculated using the lower and upper bound of the confidence interval of the exposure probabilities provided by the matrix. The number and proportion of night workers were estimated for 1982, 1990, 1999, 2007 and 2015 according to sex in all employed workers aged 15 years and over in metropolitan France. These exposure indicators for occupation or industry groups were also given by sex, usual or occasional night work. The occupation or industry choices were based according to whether the groups of industry or occupation were comparable over time because of the different classification versions used in the censuses (Additional file 2).

## Results

The number of night workers (usual and occasional) increased from 3.67 million in 1982 to 4.37 million in 2015 (15.8% vs 16.4%). During this same period, the French total workforce has increased by 15% from 23 million workers to nearly 27 million (Table 1).

**Table 1:** Number and proportion of usual and occasional night workers by sex and large industry group

	Men						Women				
Activity sector	Census year	N workers^a^	N usual night workers^b^	Proportion of usual night workers (%)^c^	N occasional night workers^b^	Proportion of occasional night workers (%)^c^	N workers^a^	N usual night workers^b^	Proportion of usual night workers (%)^c^	N occasional night workers^b^	Proportion of occasional night workers (%)^c^
Agriculture, Forestry and Fishing	1982	1,979,270	64,483	3.3 [1.8–4.9]	445,415	22.5 [16.1–29.1]	1,510,540	13,661	0.9 [0.3–1.7]	161,450	10.7 [6.6–14.9]
	1990	851,190	16,889	2.0 [1.1–3.0]	261,873	30.8 [21.3–40.4]	434,640	1040	0.2 [0.0–0.6]	95,355	21.9 [13.9–30.3]
	1999	662,960	13,844	2.1 [0.9–3.8]	228,626	34.5 [24.3–44.7]	286,060	902	0.3 [0.1–0.6]	58,529	20.5 [12.4–29.2]
	2007	561,180	24,427	4.4 [1.8–7.3]	167,959	29.9 [22.3–37.6]	246,210	3909	1.6 [0.5–3.0]	44,002	17.9 [10.9–25.4]
	2015	508,280	23,414	4.6 [2.0–7.4]	145,646	28.7 [22.0–35.4]	208,010	4058	2.0 [0.6–3.6]	34,064	16.4 [10.8–22.4]
Construction	1982	1,663,830	8227	0.5 [0.1–1.1]	86,931	5.2 [2.1–8.5]	148,500	455	0.3 [0.0–0.7]	1307	0.9 [0.2–2.0]
	1990	1,527,400	7910	0.5 [0.1–1.1]	86,521	5.7 [2.3–9.2]	149,540	405	0.3 [0.0–0.6]	1414	0.9 [0.2–2.1]
	1999	1,235,360	8769	0.7 [0.3–1.4]	72,118	5.8 [2.7–9.1]	108,850	275	0.3 [0.1–0.4]	1518	1.4 [0.3–3.0]
	2007	1,608,120	26,017	1.6 [0.9–2.6]	121,122	7.5 [4.0–11.2]	180,900	1337	0.7 [0.4–1.1]	4016	2.2 [0.8–4.1]
	2015	1,561,950	25,913	1.7 [0.8–2.7]	126,112	8.1 [4.3–12.0]	195,540	1550	0.8 [0.4–1.2]	4832	2.5 [0.9–4.5]
Manufacturing and extracting industries	1982	3,653,600	303,091	8.3 [4.8–12.2]	555,411	15.2 [8.9–21.7]	1,541,590	15,173	1.0 [0.2–2.1]	40,343	2.6 [0.6–5.2]
	1990	3,301,150	260,526	7.9 [4.6–11.6]	502,967	15.2 [9.0–21.7]	1,415,830	12,690	0.9 [0.2–1.9]	39,330	2.8 [0.7–5.5]
	1999	3,001,980	292,806	9.8 [6.1–13.8]	469,111	15.6 [9.6–21.9]	1,229,000	25,310	2.1 [0.4–4.2]	49,743	4.0 [1.1–7.5]
	2007	2,774,150	432,226	15.6 [11.3–20.1]	291,072	10.5 [5.8–15.4]	1,122,160	61,851	5.5 [2.5–9.0]	44,341	4.0 [1.1–7.4]
	2015	2,374,660	359,239	15.1 [10.9–19.6]	257,933	10.9 [6.1–15.9]	978,820	51,542	5.3 [2.3–8.7]	41,524	4.2 [1.2–7.9]
Service sector	1982	6,506,290	336,518	5.2 [2.9–7.8]	1,145,107	17.6 [12.4–23.1]	6,183,930	143,248	2.3 [1.4–3.5]	350,679	5.7 [3.3–8.4]
	1990	7,154,890	364,835	5.1 [2.9–7.6]	1,266,040	17.7 [12.3–23.4]	7,435,580	173,735	2.3 [1.4–3.4]	449,804	6.0 [3.7–8.8]
	1999	7,801,420	448,547	5.7 [3.4–8.3]	1,342,803	17.2 [12.0–22.7]	8,724,940	237,636	2.7 [1.8–3.9]	561,218	6.4 [4.0–9.2]
	2007	9,058,080	872,001	9.6 [6.7–12.8]	1,224,388	13.5 [8.9–18.4]	10,799,110	475,226	4.4 [3.0–6.1]	554,420	5.1 [2.8–7.7]
	2015	9,387,340	882,494	9.4 [6.5–12.6]	1,272,920	13.6 [8.8–18.6]	11,503,370	523,793	4.6 [3.1–6.2]	620,986	5.4 [3.0–8.1]
Total	1982	13,802,990	712,319	5.2 [2.9–7.7]	2,232,864	16.2 [10.8–21.8]	9,384,580	172,536	1.8 [1.0–2.9]	553,780	5.9 [3.4–8.8]
	1990	12,834,630	650,159	5.1 [2.9–7.6]	2,117,402	16.5 [10.9–22.4]	9,435,590	187,869	2.0 [1.1–3.0]	585,903	6.2 [3.6–9.2]
	1999	12,701,720	763,966	6.0 [3.6–8.7]	2,112,658	16.6 [11.2–22.3]	10,348,850	264,123	2.6 [1.5–3.8]	671,008	6.5 [3.9–9.5]
	2007	14,001,530	1,354,672	9.7 [6.7–12.9]	1,804,541	12.9 [8.2–17.8]	12,348,390	542,323	4.4 [2.8–6.2]	646,780	5.2 [2.8–8.0]
	2015	13,832,230	1,291,060	9.3 [6.4–12.5]	1,802,610	13.0 [8.3–18.0]	12,885,730	580,943	4.5 [2.9–6.3]	701,406	5.4 [2.9–8.3]

^a^Number of workers in the census (rounded to ten)

^b^Number of usual or occasional night workers estimated using the JEM probabilities

^c^Proportion of usual or occasional night workers and their sensitivity intervals calculated using the lower and upper bound of the confidence interval of the exposure probabilities provided by the matrix

### Men

The total number of workers in France remained relatively stable between 1982 and 2015 with a workforce estimated at 13.0 to 14.0 million men per year (Table 1). During this period, the number of usual night workers increased by 80% from 712,000 to 1.29 million, while the estimated number of occasional night workers decreased by 20% from 2.23 to 1.80 million. In 2015, the proportions of usual and occasional night workers among men represented 9.3% 95%SI[6.4–12.5] and 13.0% [8.3–18.0] of the workforce, respectively.

In 1982, the 712,000 usual night workers were primarily employed in the service sector (337,000, 47%) and in the manufacturing and extracting industries (303,000, 43%). In 2015, among the 1.29 million usual night workers the most part (882,000, 63%) was employed in the service sector and 0.36 million in the manufacturing and extracting industries (28%). During the study period, the number of occasional night workers also increased slightly in the service sector (1.14 to 1.27 million), while it decreased sharply in the manufacturing and extracting industries (5.55 to 2.58 million). Nevertheless, the latter was the industry where the proportion of usual night workers was the highest in 2015 with 15.1% (Table 1).

### Women

The contribution of women to the workforce in France increased sharply from 9.38 to 12.88 million women between 1982 and 2015 (+ 37%). In the same time, the number of usual night workers among women increased from 173,000 to 581,000 (+ 236%) and the number of occasional night workers from 554,000 to 701,000 women (+ 25%) (Table 1). In 2015, the proportions of usual and occasional night workers among women represented 4.5% [2.9–6.3] and 5.4% [2.9–8.3] of the workforce, respectively (Table 1).

The vast majority of the usual night workers among women was employed in the service sector (83% in 1982 vs 90% in 2015). This was also true for occasional night workers in 2015. The highest proportions of usual night workers in 2015 were observed in the manufacturing and extracting industries (5.3%) and in the service sector (4.6%) (Table 1).

### Occupation and industry

In the service sector, the number of workers has largely increased between 1982 and 2015 (13 million vs 21 million, + 44% in men and + 85% in women). The proportion of night workers among men was quite stable over the period (23% that represents 2 million night workers in 2015) but increased by 25% in women (1.14 million in 2015) (Table 1). However, usual night work increased from 5 to 9% in men and doubled in women over the same period. In 2015, 1.4 million workers were usually working at night and 1.9 million occasionally and 35% were women.

**Fig. 2:**
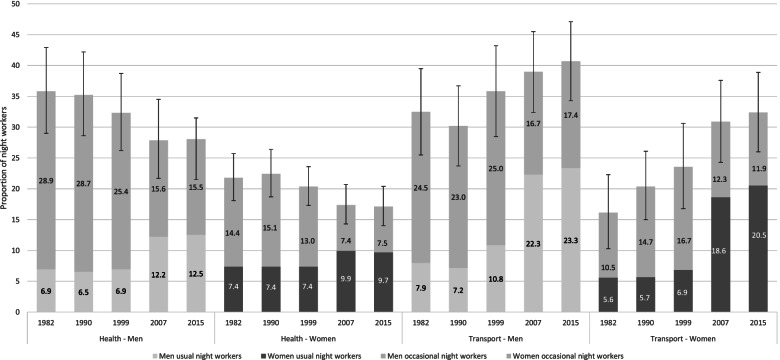
Trend in the proportion of night workers by sex. Health and transport activities The sensitivity intervals presented on the figure were calculated for the proportion of total night workers (usual + occasional night workers)

In the transport sector, which is a male dominated sector (78% men in 1982 and 62% in 2015), night work increased by 25% in men (32% in 1982 to 41% in 2015, around 25,000 night workers), and doubled in women (16 to 32%, 12,000 night workers) (Fig. 2). The proportion of usual night workers has tripled in men and almost quadrupled in women over the same period. The number of male road transport night workers stayed stable during all the studied period, but the proportion of usual night workers increased by 75% (29% in 2015) while occasional night workers decreased by 25% (Table 2). Female road transport workers are scarce compared to men (7000 women vs 300,000 men) but among them 39% worked usually at night and 9% occasionally in 2015.

**Table 2:** Number and proportion of usual and occasional night workers by sex and occupation

	Men						Women				
Occupation	Census year	N workers^a^	N usual night workers^b^	Proportion of usual night workers (%)^c^	N occasional night workers^b^	Proportion of occasional night workers (%)^c^	N workers^a^	N usual night workers^b^	Proportion of usual night workers (%)^c^	N occasional night workers^b^	Proportion of occasional night workers (%)^c^
Craftspeople bakers or pastry chefs	1982	42,810	31,327	73.2 [66.0–80.3]	5509	12.9 [7.4–18.3]	1380	61	4.4 [0.0–12.9]	156	11.3 [0.0–24.2]
	1990	38,510	28,267	73.4 [66.1–80.7]	4942	12.8 [7.3–18.4]	1840	88	4.8 [0.0–13.9]	216	11.8 [0.0–25.4]
	1999	37,980	29,230	77.0 [70.5–83.4]	5003	13.2 [8.0–18.4]	8190	328	4.0 [0.0–11.4]	624	7.6 [0.0–17.4]
	2007	34,540	27,542	79.7 [74.7–84.7]	3094	9.0 [5.4–12.5]	13,750	807	5.9 [0.9–10.9]	2115	15.4 [7.7–23.0]
	2015	30,150	24,041	79.7 [74.7–84.7]	2698	8.9 [5.4–12.5]	12,820	752	5.9 [0.9–10.9]	1971	15.4 [7.7–23.0]
Bakers or pastry chefs except industrial activities	1982	67,440	36,754	54.5 [48.8–60.2]	11,929	17.7 [13.2–22.2]	1100	86	7.9 [0.0–22.6]	108	9.8 [0.0–26.6]
	1990	62,030	33,543	54.1 [48.1–60.1]	11,117	17.9 [13.0–22.8]	2280	167	7.3 [0.0–21.0]	184	8.1 [0.0–22.6]
	1999	55,770	28,744	51.5 [46.4–56.7]	9617	17.2 [12.9–21.6]	3280	740	22.5 [14.7–29.9]	177	5.4 [0.0–15.6]
	2007	62,870	37,702	60.0 [54.2–65.7]	5280	8.4 [5.0–11.8]	7780	1246	16.0 [7.9–24.1]	227	2.9 [0.0–6.9]
	2015	60,540	36,072	59.6 [53.8–65.4]	5098	8.4 [5.0–11.9]	9550	1655	17.3 [8.6–26.1]	301	3.2 [0.0–7.4]
Salaried general care nurses	1982	14,390	3103	21.6 [13.6–29.7]	6736	46.8 [36.2–57.8]	145,280	26,133	18.0 [15.7–20.2]	44,643	30.7 [27.6–33.8]
	1990	14,280	3064	21.5 [13.2–29.9]	6631	46.4 [35.5–57.8]	174,760	31,327	17.9 [15.6–20.3]	53,434	30.6 [27.5–33.7]
	1999	24,460	4645	19.0 [12.5–25.5]	8757	35.8 [28.9–43.0]	232,460	42,692	18.4 [16.2–20.6]	59,961	25.8 [23.0–28.5]
	2007	38,860	12,407	31.9 [24.9–39.6]	7005	18.0 [11.3–25.6]	320,150	84,520	26.4 [23.4–29.4]	49,782	15.5 [12.9–18.2]
	2015	47,510	15,146	31.9 [24.1–40.7]	8329	17.5 [11.0–24.8]	360,390	92,748	25.7 [22.6–28.9]	54,874	15.2 [12.6–18.0]
Self-employed nurses	1982	2180	41	1.9 [0.0–5.5]	1071	49.0 [35.6–62.5]	18,840	77	0.4 [0.0–1.2]	7289	38.7 [32.6–44.8]
	1990	4670	88	1.9 [0.0–5.5]	2288	49.0 [35.6–62.5]	30,890	127	0.4 [0.0–1.2]	11,950	38.7 [32.6–44.8]
	1999	6280	216	3.4 [0.0–8.1]	2487	39.6 [27.0–52.2]	41,310	548	1.3 [0.0–2.6]	14,451	35.0 [29.6–40.3]
	2007	10,570	0	0.0 NA	2577	24.4 [16.1–32.7]	50,400	994	2.0 [0.6–3.3]	13,458	26.7 [22.4–31.0]
	2015	14,570	0	0.0 NA	3551	24.4 [16.1–32.7]	71,470	1410	2.0 [0.6–3.3]	19,083	26.7 [22.4–31.0]
Specialist nurses (other than psychiatric and pediatric nurses)	1982	1970	393	19.9 [2.5–37.4]	1180	59.8 [38.4–81.3]	14,940	3029	20.3 [12.2–28.4]	8082	54.1 [44.1–64.1]
	1990	3080	614	20.0 [2.5–37.5]	1843	59.9 [38.5–81.4]	18,990	3848	20.3 [12.1–28.4]	10,285	54.2 [44.1–64.2]
	1999	4390	878	20.0 [17.1–22.9]	2810	64.0 [45.2–82.8]	21,540	3652	17.0 [10.2–23.7]	12,298	57.1 [48.2–66.0]
	2007	4740	2991	63.1 [46.0–80.2]	932	19.7 [5.6–33.8]	16,870	5722	33.9 [25.1–42.8]	4993	29.6 [19.6–39.5]
	2015	5240	3304	63.1 [46.0–83.2]	1027	19.6 [5.5–33.7]	17,340	5874	33.9 [25.0–42.7]	5133	29.6 [19.6–39.5]
Midwife (employees or self-employed)	1982	20	0	0.0 NA	0	0.0 NA	9260	2381	25.7 [16.7–34.7]	4660	50.3 [40.0–60.6]
	1990	50	0	0.0 NA	0	0.0 NA	10,750	2769	25.8 [16.7–34.8]	5421	50.4 [40.1–60.8]
	1999	70	0	0.0 NA	0	0.0 NA	13,570	3028	22.3 [14.7–29.9]	5713	42.1 [33.1–51.1]
	2007	280	240	85.7 NA	0	0.0 NA	18,960	12,013	63.4 [55.3–71.4]	2787	14.7 [8.7–20.7]
	2015	680	579	85.7 NA	0	0.0 NA	22,840	14,319	62.7 [54.7–70.7]	3357	14.7 [8.8–20.6]
Army police officers (under sergeant)	1982	62,150	7024	11.3 [9.2–13.4]	51,643	83.1 [80.6–85.6]	220	17	8.0 [0.0–18.6]	190	87.8 [74.9–100.0]
	1990	63,790	7210	11.3 [9.2–13.4]	53,006	83.1 [80.6–85.6]	1440	114	8.0 [0.0–18.6]	1262	87.8 [75.1–100.0]
	1999	58,930	8134	13.8 [11.5–16.1]	46,125	78.3 [75.5–81.0]	2550	376	14.8 [3.9–25.6]	1427	56.0 [40.8–71.2]
	2007	69,310	37,827	54.6 [50.2–59.0]	25,675	37.0 [32.8–41.3]	10,950	6619	60.5 [49.8–71.1]	3236	29.6 [19.6–39.5]
	2015	57,120	31,175	54.6 [50.2–59.0]	21,156	37.0 [32.8–41.3]	11,970	7237	60.5 [49.8–71.1]	3539	29.6 [19.6–39.5]
Firefighters	1982	19,380	7966	41.1 [33.5–48.7]	9916	51.2 [43.5–58.9]	50	0	0.0 NA	24	46.2 NA
	1990	23,690	9747	41.1 [33.5–48.8]	12,127	51.2 [43.5–58.9]	140	0	0.0 NA	66	47.1 NA
	1999	29,180	9840	33.7 [27.9–39.6]	17,558	60.2 [54.1–66.2]	490	150	30.8 [0.0–66.2]	246	50.6 [12.2–89.0]
	2007	47,240	33,532	71.0 [66.3–75.7]	9679	20.5 [16.3–24.7]	1980	726	36.7 [8.5–64.8]	352	17.8 [0.0–40.1]
	2015	44,120	31,325	71.0 [66.3–75.7]	9042	20.5 [16.3–24.7]	2050	748	36.5 [8.3–64.8]	367	17.9 [0.0–40.5]
Road transport workers	1982	330,880	55,362	16.7 [13.7–19.9]	110,986	33.5 [28.7–38.4]	1310	0	0.0 NA	694	52.9 [22.0–83.8]
	1990	320,640	56,886	17.7 [14.6–21.1]	113,223	35.3 [30.4–40.2]	1280	0	0.0 NA	703	55.1 [23.6–86.6]
	1999	295,650	59,069	20.0 [17.1–22.9]	97,894	33.1 [29.2–37.0]	2560	634	24.8 [4.2–45.4]	586	22.9 [7.3–40.6]
	2007	308,170	89,041	28.9 [25.8–32.1]	77,776	25.2 [21.8–28.7]	5270	2014	38.2 [20.9–56.0]	460	8.7 [0.6–16.8]
	2015	300,690	87,531	29.1 [26.0–32.3]	75,792	25.2 [21.9–28.5]	7280	2814	38.7 [21.2–56.5]	630	8.7 [0.6–16.7]

^a^Number of workers in the census (rounded to ten)

^b^Number of usual or occasional night workers estimated using the JEM probabilities

^c^Proportion of usual or occasional night workers and their sensitivity intervals calculated using the lower and upper bound of the confidence interval of the exposure probabilities provided by the matrix

*NA* Not applicable due to less than 100 exposed workers estimated

Conversely, the health sector, which is a female dominated sector (79% women in 2015), has largely increased between 1982 and 2015 from 1.6 million workers to 3.9 million (+ 143%). On the contrary, the proportion of night workers in this sector decreased for both men and women over the same period (− 22%). However, after 1999, we observed a great increase in usual night work (+ 81% in men and + 31% in women) (Fig. 2). Self-employed nurses are mostly working occasionally at night, although we observe a decrease by 50% in men and by 45% in women over the period (Table 2). On the other hand, general care nurses (salaried nurses) are more working usually at night particularly since 2007 (32% in men, 26% in women, 103,000 workers) than by occasional night work (18% in men, 15% in women, 63,000 workers). The number of midwife night workers largely increased between 1982 and 2015 (+ 146%) even if the proportion stayed stable in women (around 75% night workers) with a high increase for usual night work after 2000 (+ 143%).

In 2015, more than 90% of male army police officers and firefighters worked at night, representing 52,000 officers and 40,000 firefighters. The proportion of these night workers stayed quite stable during the studied period. In 2015, 60% of women army police officers (11,000 working nights) and 36% of women firefighters (1000) were usually working at night.

## Discussion

This study describes the prevalence and proportion of night work among workers in France over more than 30 years, regardless of their status (salaried or self-employed), based on job-exposure matrices and census data.

Our study clearly shows an increase of usual night work in France from the 2000s (4.5% in 1999 to 7.0% in 2015). Conversely occasional night work has been less frequent (12.1% in 1999 to 9.4% in 2015) with overall night work being relatively stable over this period. The most important change concerns night work among women, who are increasingly working at night (7.7% in 1982 to 9.9% in 2015). This is explained by the French legislation concerning night work, which was until 2001 different according to sex. Before 2001, women were not allowed to work at night excepted in specific activities. According to Eurostat, nearly 2% of working French women were usual night workers in 1992 compared to nearly 5% in 2012 with an increase from 2.4 to 3.8% when considering the years framing the changes in the legislation [26].

The very large increase in the number of women working at night can also be explained by the growth in working women over this period (+ 37%) and particularly in job where women work usually at night (+ 150%). In comparison, the number of workers among men is relatively stable over the period (+ 0.2%) and usual night work increase moderately than among women (+ 79% in men). In 2015, the usual night workers are mainly in the service sector (1.4 million men and women) and in manufacturing and extracting industries (410,000 workers), the same observation applies to occasional night workers (1.9 million and 300,000 workers respectively). Night work was particularly frequent in public health activities, e.g. nurses, public administration, e.g. army officers, road transport activities, e.g. drivers, or among blue collar workers in the food-processing industries. It should be noted that the decrease in the number of night workers in the manufacturing and extracting industries could be explained by the sharp reduction of the workforce in this sector.

The Sumer surveys document the exposure of salaried workers in France to a wide range of occupational hazards. These national cross-sectional surveys were conducted in 1994, 2003, 2010 and 2017 by the French Directorate for Research, Studies and Statistics (DARES) and the French Ministry of Labour to assess occupational hazards among 25,000 to 50,000 French salaried workers based on questionnaire completed during the occupational health visits. The 2010 and 2017 surveys show that 14% of employees used to work at night between midnight and 5 AM even occasionally (20% among men and 8% among women, 3,521,100 employees working at night in 2017) [27–29]. Our own estimates for close years (2007 and 2015) were similar with 16% 95%SI [11–20] of night workers (22% [17–28] in men and 9% [6–13] in women, 3,307,100 employees working at night in 2015), despite the different exposure assessment method between the two studies. The occupations and industries with the highest number of night workers were also similar in the two studies.

At the European level, Eurostat compiles data on the active population of the Member States [26]. France is comparable in terms of percentage of night workers (usual and occasional) to the Netherlands, Finland and Greece (14.9%, 15.0 and 15.6% respectively vs 16% in France), but different from Portugal which has the lowest proportion (10%) or from Slovakia with the highest (23%). Our results are also similar to those in the United States with 9.1% [8.3–10.0] of men, and 5.6% [5.0–6.2] of women usually working at night compared to 9.3 and 4.5% respectively in our study [30]. The proportion of night work in sectors such as Healthcare and Manufacture are also comparable with 11.8%[9.6–14.6] and 10.8%[8.9–13.1] (10.3 and 12.2% respectively in our study). In 2011, in Canada, the proportion of usual night workers is higher than in France (12% vs 7% in 2015) but it includes rotating shifts [12]. However, comparisons of data at the international level must be made with caution due to variations in data collection methodology and definition of night work.

The PCS and NAF classifications used in both the Labour Force Surveys and in the Population Censuses have changed over time. The linkage between JEMs and population data could therefore be carried out based on the versions of classifications defined by period. Thus, we chose to develop several JEMs corresponding to periods with same versions of classifications, rather than developing only one JEM integrating a single version of classifications and several exposure periods. Only jobs from the 1982 and 1990 censuses coded in earlier versions of job classifications had to be cross-walked in order to be linked with the JEMs, using tables provided by the National Institute of Statistics (INSEE). This methodology reduced errors in matching the JEMs with the census data, but presents limitations for the study of temporal trends. Due to the modifications of the coding rules for certain jobs, we were unable to study the trends in night work exposure prevalence over the 30-year period in for example “Manufacture of leather and related products” (Additional file 2).

The French Labour Force survey concerns a very large sample of the population at work; however, some jobs (PCSxNAF defined at the finest level) present in the general census may not be represented in the survey and therefore not evaluated in the PCSxNAF JEMs. To limit this problem, a matrix developed on the PCS regardless of NAF (PCS JEM) was used for the jobs not assessed rather than consider them not exposed to night work (20% of the overall population was assessed by the PCS JEM in 1999). Eventually, few jobs were not evaluated (1016 individuals for the 1999 census out of more than 23 million individuals) but they are little concerned by night work (in 2007 and 2015 the unassessed PCS concern only women for field jobs in the construction sector). The changes in the frequency modality of night work rise a question about the increase observed in our results after 2000. For the entire population, we do indeed observe an increase in the percentage of usual night workers after 2000 (4.5% in 1999 vs 7% in 2015) and conversely a decrease in the percentage of occasional night workers (12.1% vs 9.4%), but this trend is also visible between 1990 and 1999. Moreover, the analysis carried out by occupational groups usually working at night before the 2003 legislation (nurses, army police officers) shows rather the opposite trend, with an increase in usual night work over time. The change in the definition of this frequency modality therefore does not seem to have had an impact on the results after 2003. The surveys after 2012 were not used in this analysis because of a new change in the question where the exposure to night work was assessed only in the last four weeks of work prior the interview and with important changes in the frequency modalities based on the percentage of work time (Additional file 1).

The JEMs for night work presented in this paper were developed using data collected in France from large samples of workers during cross-sectional surveys repeated over several decades. These data provided a solid basis for developing our job-exposure matrices using an a posteriori method [31, 32]. The large amount of data retrieved in census with detailed occupation data permits to analyse exposure to night work at a detailed level. The JEMs are easy tool that help assess exposure especially when information is not available such as night work. JEMs present some limitations such as the use of occupation and industry classifications that may group jobs with different exposures. Therefore, the JEMs exposure indices are averaged by job code and take into account the variation of exposure between different jobs or different seasons or different activities characteristics. When exposure to night work is studied as a risk factor for an outcome, it should be considered as a proxy as it does not take into account all the complex combination leading to circadian disruption [33]. Although this night work JEM is specific to the French working organization, our method is reproducible to obtain JEM specific to every working organization as similar data (census and labour force surveys) are available in many countries. This study is also easily reproducible on future data census and assess exposure to all workers in France regardless of sex and worker status (salaried and self-employed).

Although only results on night work are presented in this article, “evening” and “shiftwork” matrices has been developed using the same methodology and are available. It is also planned to develop a matrix combining night and shiftwork in order to take into account every type of shift rotation. This JEM could be used to estimate health impact in epidemiological studies (e.g. estimation of population-attributable fractions to night shift work for several cancers such as breast and prostate cancer), if additional data are available on exposures to other factors involving circadian disruptions, such as light at night, sleep disturbances, poor diet, lack of physical activity, lack of vitamin D [33–35].

## Conclusion

This study presents the trends in workers working at night usually and occasionally according to industries and occupations over 34 years in order to monitor the trend of this exposure on the entire population at work and help target the occupational groups with the highest proportion of night workers. The development of matrices has also been extended beyond 2013 and makes it possible to construct new JEMs from the future French Labour Force surveys data.

## Supplementary Information

**Additional file 1.** Detailed JEM development methodology.

**Additional file 2.** Groups of occupation and industries comparable over time throughout the different job classifications used in the Censuses.

## Data Availability

The night shift work JEMs will be available for consultation on the Exp-Pro website (http://www.exppro.fr) and the exposure indicators will be available by sex and region on the Santé publique France’ Géodes portal (http://www.geodes.santepubliquefrance.fr). The JEMs file are available upon reasonable request from Marie-Tülin Houot (marie.houot@santepubliquefrance.fr). The census data and French Labour Force surveys produced by the French National Institute for Statistics and Economic Studies (Insee) are publicly available on their website (http://www.insee.fr). The detailed files used for this study which include occupation and industry codes using French classification, sex, age, residency department, questions on night work (only for the Labour Force Survey) and number of workers are available upon request through the Insee website.
