# Supplementary material for: A 34-year overview of night work by occupation and industry in France based on census data and a sex-specific job-exposure matrix
Source: BMC Public Health. 2022 Jul 29;22:1441. doi: 10.1186/s12889-022-13830-5 (PMC9336015; doi:10.1186/s12889-022-13830-5)
Supplement: Supplementary file 1 — Additional file 1. Detailed JEM development methodology. [file 12889_2022_13830_MOESM1_ESM.pdf]

A 34-year overview of night work by occupation and industry in France based on census data and a sex-specific job-exposure matrix.

Marie-Tülin Houot[1] (marie.houot@santepubliquefrance.fr) , Nastassia Tvardik[2] (nastassia.tvardik@gmail.com), Emilie Cordina-Duverger[2] (emilie.cordina@inserm.fr), Pascal Guénel[1,2] (pascal.guenel@inserm.fr), Corinne Pilorget[1] (corinne.pilorget@santepubliquefrance.fr)

[1] Santé publique France, The French Public Health Agency, 12 rue du val d'osne 94415 Saint-Maurice, France

[2] Center for Research in Epidemiology and Population Health (CESP), Team Exposome and Heredity, Inserm, Université Paris-Saclay, Institut Gustave-Roussy 94807 Villejuif, France

Additional file 1: Detailed JEM development methodology

1. Sex-specific JEM combining PCS and NAF – PCSxNAF JEM

The job-exposure matrix (JEM) assessing exposure to night work (between midnight and 5:00 AM, definition from the French legislation) in France was developed using French national Labour Force Surveys (*"Enquête Emplois"*) from The National Institute of Statistics and Economic Studies (INSEE) from 1993 to 2012. These surveys provide, annually or continuously (from 2003), information on working hours from a sample of 110,000 to 150,000 individuals aged over 15 years, living in randomly selected households and interviewed by a home investigator. The survey question used to develop the matrix was "Do you work at night, i.e. between midnight and 5:00 AM?" asked in a specific part on the main job from 1993 to 2002 or "*In your main job, do you work at night, between midnight and 5:00 AM, usually, occasionally or never?*" from 2003 to 2012, classifying workers into three categories of night-work frequency, which will later be referred to as "Usual night workers", "Occasional night workers" and "Never night workers" (table 1). The jobs from each interviewed individual were coded, at their most detailed level, using the French classifications for occupation ("*Profession et catégorie socioprofessionnelle*", PCS code) and for activity sector ("*Nomenclature des activités française*", NAF code).

Before assessing exposure based on these surveys, the data was managed to keep only one record for each individual (interviewed from the first quarter of the year for surveys from 2003 to 2012) and complete records on the PCS and NAF codes and night work. In order to have enough power for the analysis, the survey data were grouped by 5-year period (1993-1997, 1998-2002, 2003-2007, 2008-2012).

Then, to assess the exposure using the PCS and the NAF codes, several steps were implemented:

- Exposure proportions to night work (usual and occasional) were calculated according to the pair PCS code-NAF code at two levels of aggregation:
    - 4-digit PCS code (PCS4) combined with 3-digit NAF code (NAF3),
    - PCS4 combined with 2-digit NAF code (NAF2)
- For each level of aggregation, the precision of the proportion (Δ) was calculated in groups with more than 30 subjects using $\Delta = 1{,}96 * \sqrt{\frac{pq}{n}}$ with p: proportion of exposed (night workers), q: proportion of non-exposed, n: total number in the group.
- For each pair PCS4xNAF3, an exposure probability (the proportion of night workers) was assigned from the most detailed level when the precision of the proportion (Δ) was lower than 10%, otherwise the PCS4xNAF2 exposure probability was assigned using the same precision criteria. If the precision criteria could not be reached at the PCS4xNAF2 level, no exposure probability was assigned to the pair.
- Then according to the level chosen previously, the exposure proportion in men and women and their confidence intervals were calculated and assigned to each pair PCSxNAF.

2. Sex-specific JEM PCS regardless of NAF – PCS JEM

As the PCSxNAF JEM did not assess every PCSxNAF pair due to low number of workers in certain pairs and also because the French national Labour Force Surveys (*"Enquête Emplois"*) did not include all jobs retrieved in the French Census, we developed another JEM based only on the PCS code regardless of the NAF using the same methodology presented in paragraph A.1.

The exposure was assessed through the same steps than previously:

- Exposure proportions to night work (usual and occasional) were calculated according to PCS4 regardless of a NAF code at two levels of aggregation:
    - PCS4 not combined with a NAF code
    - PCS3 not combined with a NAF code
- For every level of aggregation, the precision of the proportion (Δ) was calculated in groups with more than 30 subjects using $\Delta = 1{,}96 * \sqrt{\frac{pq}{n}}$ with p: proportion of exposed, q: proportion of non-exposed, n: total number in the group.
- For each PCS4, an exposure probability (the proportion of night workers) from the PCS4 aggregation was assigned if the precision was lower than 10%, otherwise the exposure probability from PCS3 aggregation was assigned.
- Then according to the level chosen previously, the exposure proportion in men and women and their confidence intervals were calculated and assigned to each PCS.

Table 1: Data description for night work of the annual French surveys between 1993 and 2012.

Period	1					2					3					4					5				
**Survey's year**	**1993**	**1994**	**1995**	**1996**	**1997**	**1998**	**1999**	**2000**	**2001**	**2002**	**2003**	**2004**	**2005**	**2006**	**2007**	**2008**	**2009**	**2010**	**2011**	**2012**	**2013**	**2014**	**2015**	**2016**	**2017**
PCS version	1982										2003														
NAF version	1993										2003					2008									
Question about night work	In a specific part on the main job:<br>Do you work at night?<br>(between midnight and 5 AM)<br>1. Usually<br>2. Only some nights<br>3. Never										In your main job, do you work at night?<br>(between midnight and 5 AM)<br>1. Usually<br>2. Occasionally<br>3. Never										During those weeks, did you work at night that is to say between midnight and 5 AM?<br>1. Yes, half of my working hours or more<br>2. Yes, less than half of my working hours<br>3. No				

Help to read the table: For period 3, which includes the years 2003 to 2007, the occupations and industries in the survey were coded using the 2003 PCS classification and the 2003 NAF classification respectively. For this same period, the question about night work in the surveys was "In your main job, do you work at night?". For period 4, which includes the years 2008 to 2012, the occupations and industries were coded using the 2003 PCS classification and the 2008 NAF classification respectively. The question about night work in the surveys was "In your main job, do you work at night?".
