# Supplementary material for: A 34-year overview of night work by occupation and industry in France based on census data and a sex-specific job-exposure matrix
Source: BMC Public Health. 2022 Jul 29;22:1441. doi: 10.1186/s12889-022-13830-5 (PMC9336015; doi:10.1186/s12889-022-13830-5)
Supplement: Supplementary file 2 — Additional file 2. Groups of occupation and industries comparable over time throughout the different job classifications used in the Censuses. [file 12889_2022_13830_MOESM2_ESM.pdf]

A 34-year overview of night work by occupation and industry in France based on census data and a sex-specific job-exposure matrix.

Marie-Tülin Houot[1] (marie.houot@santepubliquefrance.fr) , Nastassia Tvardik[2] (nastassia.tvardik@gmail.com), Emilie Cordina-Duverger[2] (emilie.cordina@inserm.fr), Pascal Guénel[1,2] (pascal.guenel@inserm.fr), Corinne Pilorget[1] (corinne.pilorget@santepubliquefrance.fr)

[1] Santé publique France, The French Public Health Agency, 12 rue du val d'osne 94415 Saint-Maurice, France

[2] Center for Research in Epidemiology and Population Health (CESP), Team Exposome and Heredity, Inserm, Université Paris-Saclay, Institut Gustave-Roussy 94807 Villejuif, France

Additional file 2: Groups of occupation and industries comparable over time throughout the different job classifications used in the Censuses

1. **NAF codes used for each group of industry**
    a. **Large industry**

**NAF2008**	**NAF1993**
**Agriculture, Forestry and Fishing**	**Agriculture, Forestry and Fishing**
01-03	01-05
**Construction**	**Construction**
41-43	45
**Manufacturing and extracting industries**	**Manufacturing and extracting industries**
05-39	10-41
**Tertiary sector**	**Tertiary sector**
45-99	50-99

b. **Specific industry**

<table>
<tr><th>NAF2008</th><th>NAF1993</th></tr>
<tr><td>Health and social work activities</td><td>Health and social work activities</td></tr>
<tr><td>86 - Human health activities</td><td rowspan="3">85 - Health and social work (except 85.2Z)</td></tr>
<tr><td>87 - Residential care activities</td></tr>
<tr><td>88[i] - Social work activities without accommodation</td></tr>
<tr><td>75.00Z – Veterinary activities</td><td>85.2Z – Veterinary activities</td></tr>
<tr><td>Transport</td><td>Transport</td></tr>
<tr><td>Air transport</td><td>Air transport</td></tr>
<tr><td>51.10Z - Passenger air transport
51.21Z - Freight air transport</td><td>62.1Z – Scheduled air transport
62.2Z - Non-scheduled air transport</td></tr>
<tr><td>Water transport</td><td>Water transport</td></tr>
<tr><td>50.10Z - Sea and coastal passenger water transport
50.20Z - Sea and coastal freight water transport
50.30Z - Inland passenger water transport
50.40Z - Inland freight water transport
52.22Z - Service activities incidental to water transportation</td><td>61.1A - Sea transport
61.1B – Coastal water transport
61.2Z – Inland water transport
63.2C - Other supporting water transport activities</td></tr>
</table>

c. **Example of a specific industry that could not be studied over the period**

NAF2008	NAF1993
**Manufacture of leather and related products**	**Manufacture of leather and leather products**
15.20Z - Manufacture of footwear<br>16.29Z – Manufacture of other products of wood; manufacture of articles of cork, straw and plaiting materials<br>22.19Z – Manufacture of other rubber products<br>22. 29B – Manufacture of consumer products from plastics<br>32.30Z – Manufacture of sports goods	19.3Z – Manufacture of footwear

2. PCS codes used for each group of occupations

PCS2003	PCS1982
431a – Nursing executives	4311 - Nursing executives
431c – Nursery nurses	4313 – Nursery nurses
431d – Specialist nurses (other than psychiatric and pediatric nurses)	4314 - Specialist nurses (other than psychiatric and pediatric nurses)
431e – Midwife (employees or self-employed)	4321 – Midwife (employees or self-employed)
431f – Salaried general care nurses[ii]	4315 – Salaried general care nurses
431g - Self-employed nurses	4316 – Self-employed nurses
532a - Army police officers (under sergeant)	5312 – Army police officers (under sergeant)
533a - Firefighters	5315 - Firefighters
641a – Road transport workers	6411 - Road transport workers
642a – Taxi drivers (salaried)	6413 - Taxi drivers (salaried)
215a – Craftspeople bakers or pastry chefs from 0 to 9 employees	2101 – Craftspeople bakers or pastry chefs from 0 to 2 employees<br>2102 – Craftspeople bakers or pastry chefs from 3 to 9 employees
636c - Bakers or pastry chefs except industrial activities	6353 - Bakers or pastry chefs except industrial activities
626c : Skilled operators and workers in the heavy timber and paper-cardboard manufacturing industries	6264 : Skilled workers in the manufacture of paper and cardboard
673b : Unskilled production workers working by metal forming	6722 : Unskilled workers working by forming metal

[i] also includes a match between 88.99B (NAF2008) and 75.2A (NAF1993) for the contribution to international civil interventions of a humanitarian nature

[ii] also includes in PCS2003 “Salaried rehabilitation specialists (only for occupational therapists and pedicure nurses) and dieticians” that are not included in PCS1982.
